# A juxtacrine/paracrine loop between C-Kit and stem cell factor promotes cancer stem cell survival in epithelial ovarian cancer

**DOI:** 10.1038/s41419-019-1656-4

**Published:** 2019-05-28

**Authors:** Elena Laura Mazzoldi, Simona Pavan, Giorgia Pilotto, Kevin Leone, Anna Pagotto, Simona Frezzini, Maria Ornella Nicoletto, Alberto Amadori, Anna Pastò

**Affiliations:** 1 Veneto Institute of Oncology IOV-IRCCS, Padua, Italy; 2 Innate Pharma, Marseille, France; 3 Department of Surgery, Oncology and Gastroenterology, University of Padua, Padua, Italy; 4 Present Address: Department of Inflammation and Immunology, Humanitas Clinical and Research Center, Rozzano, Milan Italy

**Keywords:** Cancer stem cells, Ovarian cancer

## Abstract

Receptors tyrosine kinase (RTK) enable normal and tumor cells to perceive and adapt to stimuli present in the microenvironment. These stimuli, also known as growth factors, are important molecular cues actively supporting cancer stem cell (CSC) self-renewal and viability. Since in epithelial ovarian cancer (EOC) the expression of c-Kit (CD117) has been identified as a CSC hallmark, we investigated the existence of a tumor growth-promoting loop between c-Kit and its ligand Stem Cell Factor (SCF). SCF exists as a soluble or transmembrane protein and through c-Kit interaction regulates cell viability, proliferation, and differentiation both in physiological and pathological conditions. High amounts of SCF were found in the ascitic effusions collected from EOC patients. While tumor cells and CSC only expressed the membrane-associated SCF isoform, both secreted and membrane-bound isoforms were expressed by tumor-associated macrophages (TAM, here shown to be M2-like) and fibroblasts (TAF). Circulating monocytes from EOC-bearing patients and healthy donors did not express both SCF isoforms. However, monocytes isolated from healthy donors produced SCF upon in vitro differentiation into macrophages, irrespectively of M1 or M2 polarization. In vitro, both SCF isoforms were able to activate the Akt pathway in c-Kit^+^ cells, and this effect was counteracted by the tyrosine kinase inhibitor imatinib. In addition, our results indicated that SCF could help c-Kit^+^ CSC survival in selective culture conditions and promote their canonical stemness properties, thus indicating the possible existence of a juxtacrine/paracrine circuit in EOC.

## Introduction

Epithelial ovarian cancer (EOC) is the fifth leading cause of cancer-related death among females, and the first cause of death if considering only the gynecological malignancies^1^. This lethality is mainly due to the lack of specific symptoms and an efficacious screening program; as a result, EOC is usually diagnosed at advanced stages^2^. EOC is often considered chemosensitive, with a response rate higher than 80% after first-line chemotherapeutic treatments. However, about 70% of patients relapse within 18 months^3^. A wealth of data demonstrates that one of the causes for frequent tumor relapse could be ascribed to a small population of tumor-initiating cells, cancer stem cells (CSC), which represent a key element in tumor maintenance and expansion. CSC, documented in almost all tumor histotypes^4–6^, are characterized by the expression of stemness-associated genes, and by the ability to grow and generate spheroids when cultured in appropriate serum-free conditions^7,8^. Most importantly, compared with the bulk of malignant cells, CSC are endowed with a very high tumorigenic potential, and are able to generate, in immunodeficient animals, tumors which faithfully recapitulate the parental tumor^9–11^. Ovarian CSC are characterized by special properties that allow their preferential survival over the rest of bulk tumor cells. Indeed, others and we reported that CSC resist glucose starvation and do not use Warburg effect, rather privileging OXPHOS respiration, which guarantees higher energetic efficiency and protection from excessive oxygen radical production^10,12^. In addition, we showed that CSC display higher autophagic activity, compared with the bulk of differentiated tumor cells^13^. Others and we have demonstrated that CSC from EOC can be identified by the co-expression of CD44 and c-Kit (CD117), a tyrosine kinase receptor^10,14,15^.

The c-Kit ligand Stem Cell Factor (SCF) is a type II homodimer of two four-helix bundles which plays a key role as a growth factor by promoting and regulating cell viability, proliferation, and differentiation in several biological processes^16^. Due to mRNA alternative splicing involving exon 6, SCF can be expressed in either membrane-bound or soluble form. Indeed, when exon 6 is translated (SCF 248), SCF is produced as a transmembrane molecule with a cleavage site for the release of the soluble growth factor by membrane proteases. On the contrary, the mRNA variant without exon 6 (SCF 220) translates a protein lacking the cleavage site, and the molecule eventually remains membrane-bound^17^. Both the soluble and membrane-anchored SCF, after binding c-Kit, lead to downstream activation of a variety of signal transduction pathways including Ras/Erk, Jak/STAT, and PI3K, which in turn phosphorylates and activates Akt, thus promoting cell survival and proliferation^18^. Indeed, the intracellular domains of c-Kit dominate a number of cellular functions, including proliferation, cell adhesion, differentiation, and chemotaxis. While in physiological conditions SCF is mainly produced by fibroblasts and endothelial cells^19–22^, in pathological settings SCF production may originate from different sources, including tumor tissue^23,24^. It is known that SCF and its cognate receptor may also play a central role in several tumor settings^25–27^. In particular, in gastrointestinal stromal tumors (GIST), c-Kit mutations represent the driver event involved in tumor generation and maintenance^27,28^. In this context, competition of imatinib mesylate (Gleevec) with the ATP-binding pocket of c-Kit nullifies the constitutive signaling of this receptor^29^. Activating mutations aside, c-Kit expression has been occasionally described in other tumor histotypes, including breast^30^, lung^24^, glioma^31^, colon^32,33^, leukemia^34^, and gynecological cancer^35,36^, and the existence of a putative autocrine/paracrine SCF/c-Kit circuit promoting cancer outgrowth has been advanced^37–42^.

In search of the mechanisms fostering CSC survival and expansion, we addressed the role of the SCF/c-Kit axis in primary samples of ascitic effusions collected from EOC-bearing patients. We demonstrated ex vivo that a complex SCF/c-Kit juxtacrine/paracrine circuit involving stromal, immune, and cancer cells is present in EOC patients, and could be involved in promoting stemness properties of ovarian CSC.

## Results

### SCF growth factor is expressed by tumor-associated macrophages and fibroblasts

**Fig. 1:**
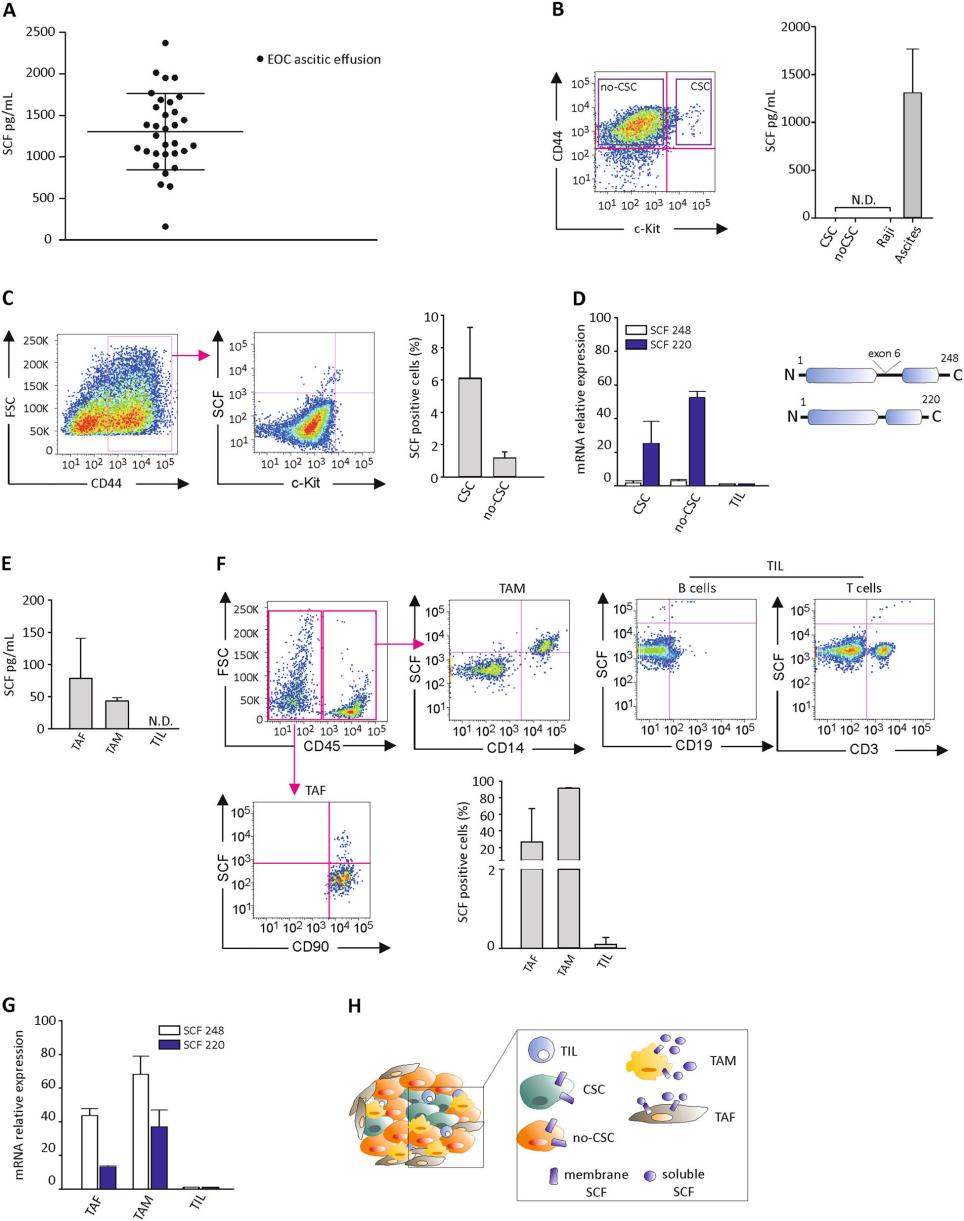
TAM and TAF from EOC patients produce soluble SCF. **a** ELISA analysis of SCF levels in ascitic effusions collected from EOC patients (*N* = 32). **b** ELISA analysis of SCF performed on conditioned media of FACS-sorted CD44^+^c-Kit^+^ (CSC) and CD44^+^c-Kit^−^ (no-CSC) cells from primary samples of EOC ascitic effusions. Raji cells and ascites from EOC patients were used as negative and positive control, respectively. The bars represent the mean ± S.D. (*N* = 4). N.D. = not detectable. Sorting gating strategy is shown on the left. **c** Flow cytometry analysis of SCF on CSC and no-CSC from primary samples of EOC ascitic effusions. The bars represent the mean ± S.D. (*N* = 4). Gating strategy is shown on the left. **d** qRT-PCR analysis of SCF 248 and SCF 220 isoforms in FACS-sorted CSC and no-CSC from EOC ascitic effusions. Data were normalized to tumor infiltrating lymphocytes (TIL). The bars represent the mean ± S.D. (*N* = 4). Schematic representation of SCF isoforms is shown. **e** ELISA analysis of SCF on conditioned media of TAF, TAM and TIL sorted by FACS from primary samples of EOC ascitic effusions. Data are expressed as mean ± S.D. (*N* = 4). N.D. = not detectable. **f** Flow cytometry analysis of SCF on TAF, TAM, and TIL from primary samples of EOC ascitic effusions. The bars represent the mean ± S.D. (*N* = 4). Gating strategy is shown. **g** qRT-PCR analysis of SCF 248 and SCF 220 isoforms in FACS-sorted TAF, TAM, and TIL from EOC ascitic effusions. Data were normalized to TIL. The bars represent the mean ± S.D. (*N* = 4). **h** Schematic representation of SCF isoform expression in the different cell subsets within the EOC microenvironment

Since SCF exists in two molecular isoforms^17^, as mentioned above, we first investigated the presence of the soluble growth factor in ascitic effusions collected from 32 EOC-bearing patients. As shown in Fig. 1a, detectable amounts of soluble SCF were found ranging from 161.83 to 2374.88 pg/mL (mean 1306.8 ± 460.5 pg/mL). No correlation between tumor histology/grading or stage and ascitic fluid SCF content was observed (Supplementary Table 1). We thus wondered whether ovarian tumor cells could contribute to these SCF levels, and whether SCF production could be a distinctive feature of the CSC subset. To this end, we evaluated the expression of the soluble SCF form in ex vivo FACS-sorted stem (CD44^+^c-Kit^+^) and non-stem (CD44^+^c-Kit^−^) cells from EOC ascitic effusion samples. When the two cell populations were cultured for 24 h, SCF could not be detected in the supernatant of either population (Fig. 1b). However, SCF positivity was recorded by flow cytometry analysis in both unsorted cell subsets, accounting for 6.1 ± 3.1% and 1.2 ± 0.4%, in CD44^+^c-Kit^+^ and CD44^+^c-Kit^−^ cells, respectively (Fig. 1c). Accordingly, as shown in Fig. 1d, FACS-sorted CD44^+^c-Kit^+^ and CD44^+^c-Kit^−^ cells only expressed mRNA for the membrane-associated SCF isoform (SCF 220).

Since EOC ascitic effusions also include tumor-associated fibroblasts (TAF) and cells of myeloid (tumor-associated macrophages, TAM) and lymphoid (tumor associated lymphocytes, TIL) origin, we evaluated SCF expression in these cell populations. As shown in Fig. 1e, detectable SCF amounts were found in the supernatants of in vitro cultured FACS-sorted TAF and TAM (78.52 ± 62.21 pg/mL and 42.90 ± 5.29 pg/mL, respectively), while no SCF release from TIL was observed. Flow cytometry analysis also demonstrated the expression of the membrane-bound SCF isoform by TAF and TAM (26.8 ± 9.9% and 91.3 ± 0.78%, respectively), but not by TIL (Fig. 1f). These results were further confirmed by qRT-PCR analysis. As expected, TAF and TAM expressed both SCF isoforms at high levels, while no SCF expression was recorded in TIL (Fig. 1g). The behavior of the different cell populations within the EOC microenvironment in terms of SCF production is summarized in Fig. 1h.

### Monocyte-to-macrophage differentiation induces SCF expression, irrespective of M1/M2 polarization

**Fig. 2:**
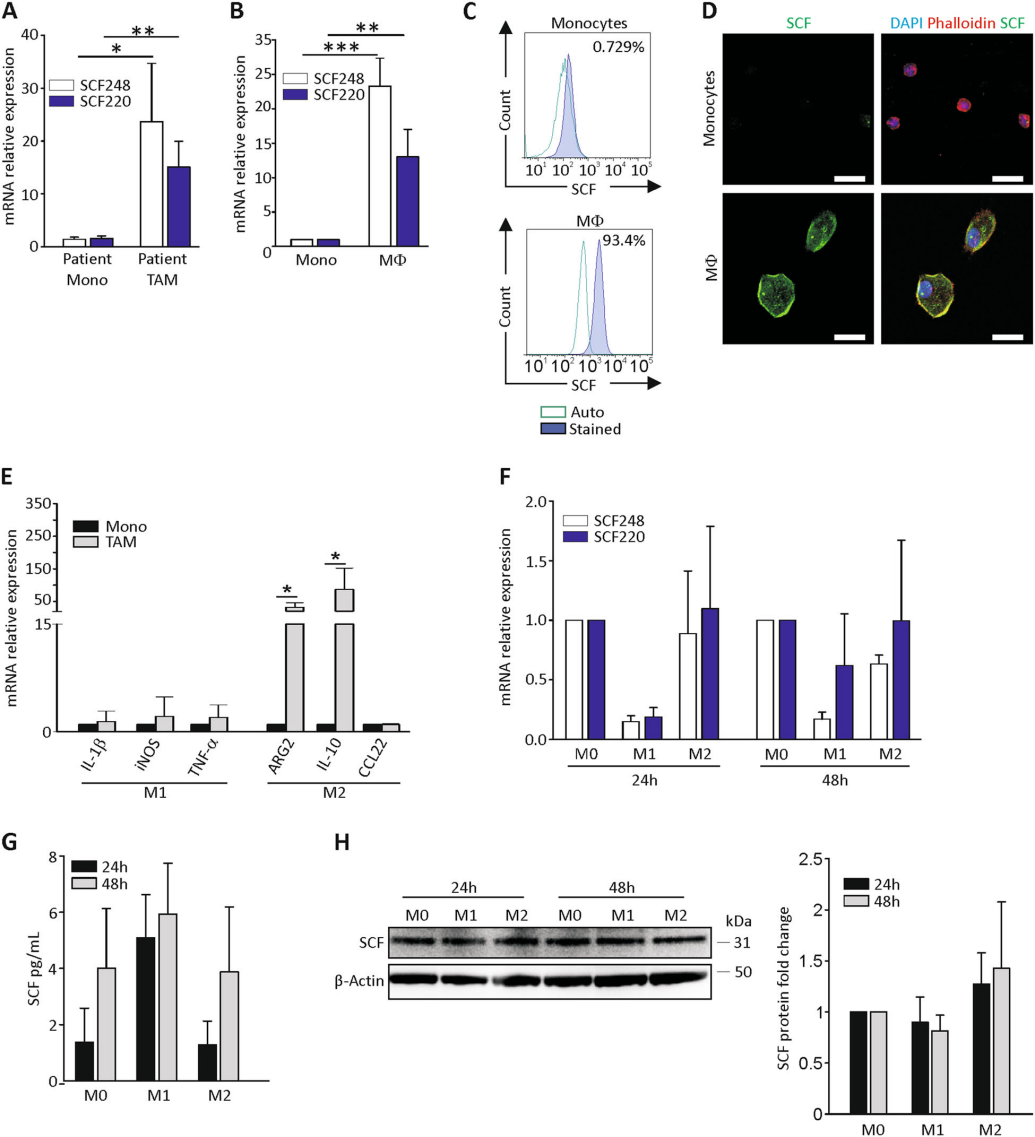
Macrophage differentiation is associated with SCF expression. **a** qRT-PCR analysis of both SCF isoforms in CD14^+^ monocytes (Mono) and TAM isolated, respectively, from peripheral blood or ascitic effusion of EOC-bearing patients. The bars represent the mean ± S.D. (*N* = 3). **b** qRT-PCR analysis of both SCF isoforms in CD14^+^ monocytes (Mono) isolated from the peripheral blood of healthy donors and macrophages (Mφ) obtained after 1 week of in vitro differentiation. The bars represent the mean ± S.D. (*N* = 3). **c**, **d** Representative flow cytometry and immunofluorescence analysis, respectively, of SCF expression in CD14^+^ monocytes isolated from the peripheral blood of healthy donors and macrophages obtained after 1 week of in vitro differentiation. In **d**, color legend: nuclei are blue (DAPI), actin is red (Phalloidin), and SCF is green. Scale bar 20 µm. **e** qRT-PCR analysis of M1 (IL-1β, iNOS, and TNF-α) and M2 (ARG2, IL-10, and CCL22) polarization markers in FACS-sorted TAM from EOC ascitic effusions. Data were normalized to monocytes. The bars represent the mean ± S.D. (*N* = 5). **f** qRT-PCR analysis of SCF 220 and SCF 248 in vitro differentiated M0, M1 and M2 macrophages after 24 h and 48 h from polarization. Data were normalized to the corresponding M0. The bars represent the mean ± S.D. (*N* = 3). **g** ELISA analysis of SCF on conditioned media of M0, M1, and M2 cells after 24 and 48 h from polarization. The bars represent the mean ± S.D. (*N* = 3). **h** WB analysis of SCF in in vitro differentiated M0, M1, and M2 macrophages after 24 h and 48 h from polarization. A representative blot is shown. SCF signal was normalized to β-actin. The bars represent the mean ± S.D. (*N* = 3). *p < 0.05, **p < 0.01, ***p < 0.001

Since SCF production by TAF was not unexpected, according to the literature^21^, we focused on TAM, the main orchestrators of the tumor inflammatory environment^43^. qRT-PCR analysis revealed that TAM isolated from EOC ascitic effusions presented higher mRNA expression levels of both SCF isoforms, compared with CD14^+^ monocytes isolated from patients’ peripheral blood (Fig. 2a). Accordingly, macrophages differentiated in vitro from healthy donor-derived monocytes acquired the expression of both the membrane-associated and complete SCF isoforms (Fig. 2b). These results were also confirmed at the protein level by cytofluorimetric and confocal analysis of resting and in vitro activated monocytes (Fig. 2c, d).

It is known that macrophages can exist as two different functional populations, namely M1 and M2. M1 (or classically activated) macrophages are characterized by an inflammatory phenotype, whereas M2 (or alternatively activated) macrophages create an immunosuppressive and tumor permissive state that may support CSC self-renewal and promote tumor growth^44^. Therefore, we investigated the expression of canonical M1 (IL-1β, iNOS, and TNF-α) and M2 (Arginase-2, IL-10, and CCL22) markers in FACS-sorted TAM from ascitic effusions, and found a polarization towards the M2 phenotype (Fig. 2e), corroborating the idea that M2 macrophages may support a unique microenvironment that protects and promotes CSC functions^44^. We thus wondered whether SCF expression could be a preferential property of the M2 subset. For this purpose, we differentiated in vitro macrophages (M0) from circulating monocytes isolated from healthy donors. After complete polarization towards the M1 or M2 phenotypes (demonstrated by TNF-α and IL-1β expression for M1 and CCL22 and IL-10 for M2; Supplementary Fig. S1), we investigated SCF expression by qRT-PCR, ELISA, and Western blot analysis. As shown in Fig. 2f, at 24 and 48 h M1 polarization seemed to be associated with a reduced expression of both SCF isoforms at the mRNA level. On the contrary, no differences were detected among the macrophage populations when growth factor release (Fig. 2g) and the total protein level (Fig. 2h) were analyzed. A possible explanation for this apparent discrepancy could be the late time point we considered and the mRNA induction kinetics. Indeed, at an earlier time point (6 h) from polarization, M1 and M2 macrophages showed comparable mRNA levels of both SCF isoforms (Supplementary Fig. S2). The peak was followed by a decrease in SCF expression that was more accentuated in the M1 subset, consistently with what reported in Fig. 2f. Altogether, these results suggest that SCF production is a property of activated macrophages, irrespective of their M1/M2 polarization.

### Both SCF isoforms activate the PI3K/Akt pathway in c-Kit^+^ cells through c-Kit phosphorylation

**Fig. 3:**
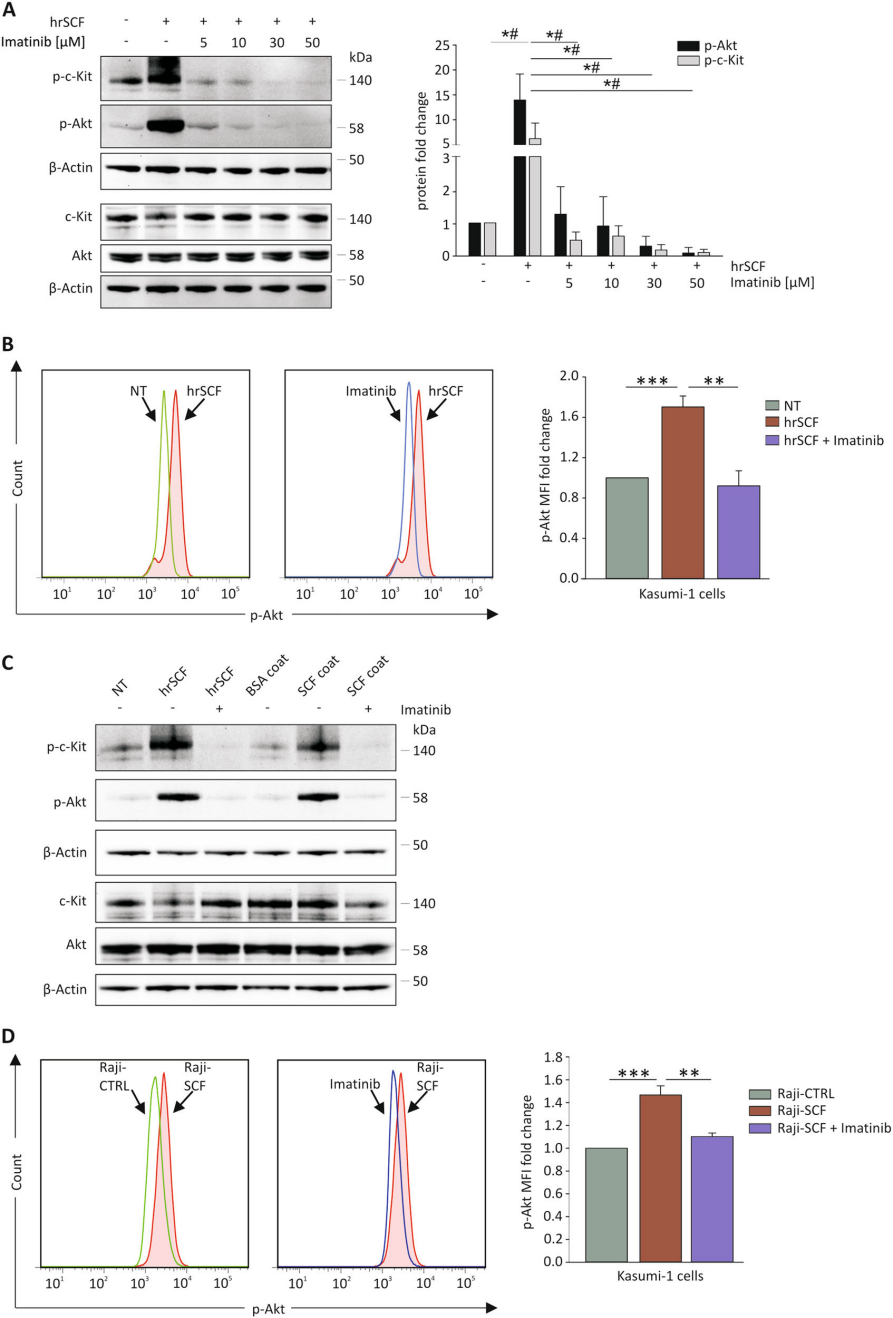
Soluble and membrane-associated SCF activate Akt signaling through the interaction with c-Kit. **a** WB analysis of p-c-Kit and p-Akt in Kasumi-1 cells pretreated or not with imatinib (5, 10, 30, and 50 μM) and stimulated with hrSCF (50 ng/mL) for 5 min. Signals were normalized to β-actin. A representative blot is shown. The bars represent p-c-Kit/c-Kit and p-Akt/Akt mean ± S.D. (*N* = 3). *, #*p* < 0.05 for p-Akt, and p-c-Kit, respectively. **b** Flow cytometry analysis of p-Akt in Kasumi-1 cells pre-treated or not with imatinib (30 μM) and stimulated with hrSCF (50 ng/mL) for 5 min. Representative FACS plots are shown. The bars represent the mean ± S.D. (*N* = 3). **c** WB analysis of p-c-Kit and p-Akt in Kasumi-1 cells pretreated or not with imatinib (30 μM) and stimulated with hrSCF (50 ng/mL) or hrSCF-coated wells for 5 min. Signals were normalized to β-actin. A representative blot out of 3 independent experiments is shown. **d** Flow cytometry analysis of p-Akt in Kasumi-1 cells pretreated or not with 30 μM imatinib and co-cultured for 5 min with Raji-CTRL or Raji overexpressing SCF (Raji-SCF). Representative FACS plots are shown. The bars represent the mean ± S.D. (*N* = 3). **p < 0.01; ***p < 0.001

Next, we wondered whether soluble and membrane SCF could activate a signaling cascade in c-Kit^+^ cells. Because of the technical hurdles in obtaining a sufficient number of vital CD44^+^c-Kit^+^ CSC from primary EOC samples by FACS sorting, we decided to exploit as a readout Kasumi-1 cells, a human leukemic cell line uniformly expressing c-Kit. Cells were treated for 5 min with human recombinant SCF (hrSCF) after a pre-treatment with scalar doses of the tyrosine-kinase inhibitor imatinib^45^. WB analysis revealed that hrSCF stimulation induced a strong c-Kit and Akt phosphorylation (Fig. 3a). Interestingly, upon hrSCF incubation, total c-Kit was downregulated (Fig. 3a), most probably as a consequence of the receptor internalization and degradation, a well-known tyrosine-kinase inactivation mechanism^16^. As expected, pre-treatment with imatinib prevented hrSCF-induced c-Kit and Akt phosphorylation in a dose-dependent manner (Fig. 3a). WB results were corroborated by flow cytometry analysis of p-Akt in Kasumi-1 cells (Fig. 3b), confirming that hrSCF stimulation increased p-Akt levels, whereas treatment with imatinib prior to hrSCF stimulation efficiently blocked Akt phosphorylation.

Similarly, we checked whether the membrane-associated SCF isoform could also efficiently induce Akt phosphorylation. To this end, we coated the plastic surface of a multiwell plate with hrSCF to mimic the membrane-associated isoform, and measured Akt phosphorylation in Kasumi-1 cells after interaction with the well bottom. As shown in Fig. 3c, a clear p-Akt band was observed in Kasumi-1 cells incubated for 5 min in hrSCF-coated wells. To better investigate the interaction of c-Kit with its membrane-bound ligand, we engineered Raji cells, a human B-lymphoma cell line negative for either SCF isoform^35^, with the non-cleavable SCF 220 isoform, tagged with GFP (Raji-SCF). Raji-CTRL cells expressing only GFP were used as a control (Supplementary Fig. S3a, b). When Kasumi-1 and transduced Raji cells were co-cultured, flow cytometry analysis demonstrated that 5 min co-culture with Raji-SCF induced a significant increase of p-Akt levels in Kasumi-1 cells (Fig. 3d), compared with Kasumi-1 cells incubated with the appropriate control. As expected, pretreatment with imatinib reverted p-Akt levels to the baseline (Fig. 3d). Altogether, these results indicate that both soluble and membrane-associated SCF are effective in activating Akt signaling through c-Kit phosphorylation.

### SCF stimulation affects the in vitro stemness properties of ovarian CSC

**Fig. 4:**
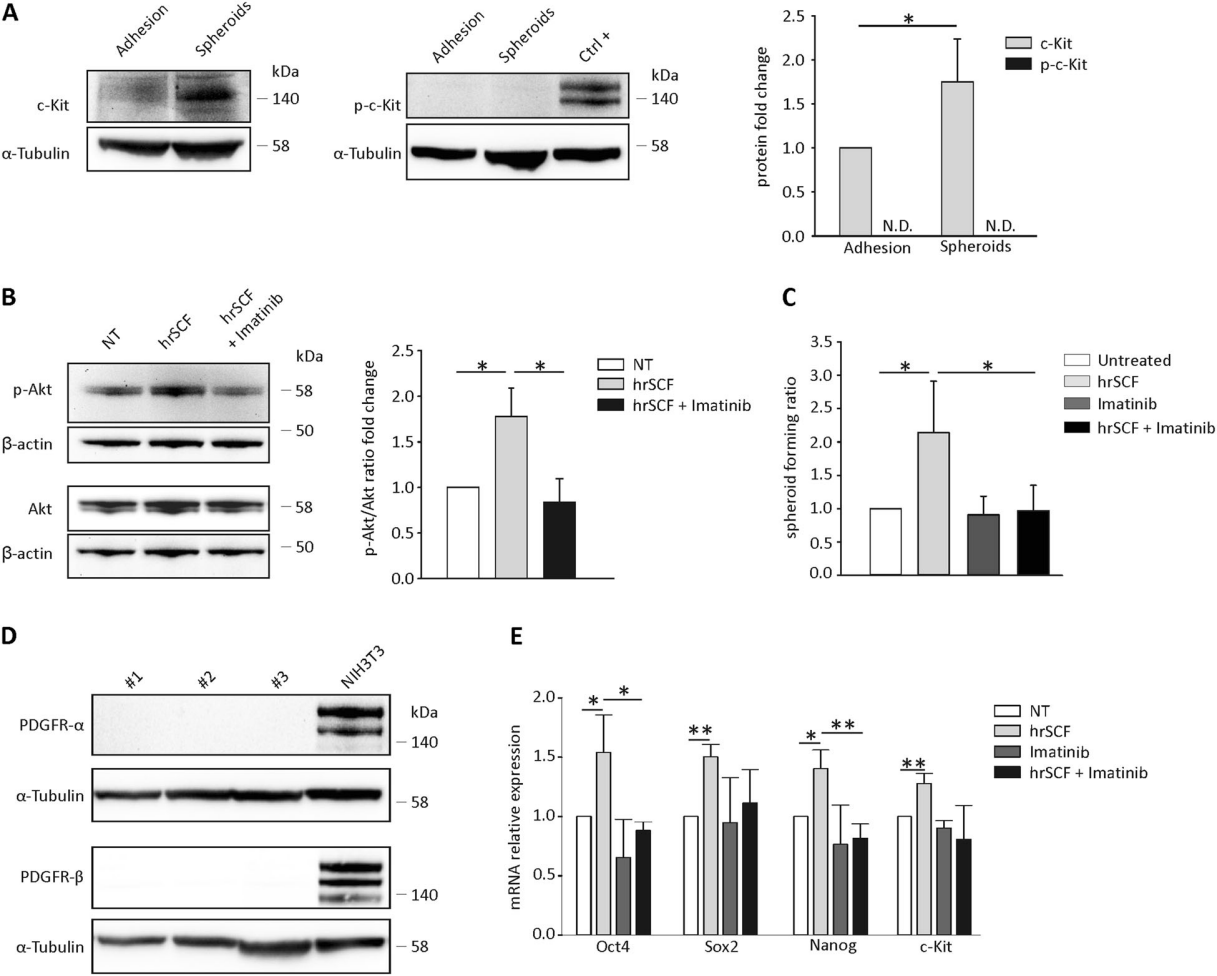
SCF modulates EOC stemness features. **a** Representative WB analysis of c-Kit and p-c-Kit expression in EOC cells cultured either under adherent or spheroid-forming conditions. Kasumi-1 cells stimulated for 5 min with SCF were used as positive control for p-c-Kit (ctrl + ). The bars represent the mean ± S.D. (*N* = 4). N.D. = not detectable. **b** WB analysis of p-Akt in EOC cells cultured under spheroid-forming conditions pretreated or not with imatinib (30 μM) and stimulated with hrSCF (50 ng/mL) for 5 min. Signals were normalized to β-actin. A representative blot is shown. The bars represent p-Akt/Akt mean ± S.D. (*N* = 3). **c** ELDA performed on EOC cells cultured for two weeks under spheroid-forming conditions in the presence of hrSCF (50 ng/mL), imatinib (5 μM) or the combination of the two. The bars represent the mean ± S.D. (*N* = 3). **d** Representative WB analysis of PDGFR-α and β in EOC samples. NIH3T3 were used as positive control. **e** qRT-PCR analysis of Oct4, Sox2, Nanog, and c-Kit mRNA levels in EOC cells cultured for two weeks as described in **a**. The bars represent the mean ± S.D. (*N* = 3). *p < 0.05, **p < 0.01

We finally wondered whether SCF stimulation could be able to affect the canonical CSC properties. One of the most recognized hallmarks of CSC is their ability to form spheroids when cultured under appropriate stemness conditions^46,47^. To this purpose, we isolated tumor cells from the ascitic fluid of patients diagnosed with serous EOC (as described in Materials and Methods). After two weeks in spheroid-forming conditions, we first verified that c-Kit expression increased compared with the adherent counterpart (Fig. 4a). Similar to Kasumi-1 cells, spheroid treatment with hrSCF induced a significant increase in p-Akt levels, which was abolished by imatinib pretreatment (Fig. 4b).

We thus tested the functional effects of hrSCF on EOC ascitic effusion cells in a spheroid-forming assay readout. Extreme limiting dilution assay (ELDA) showed that hrSCF significantly increased spheroid-forming ratio (Fig. 4c) compared with untreated cells. Imatinib alone did not exert any effect, an expected finding since c-Kit was not phosphorylated in the absence of stimuli (Fig. 4a). On the contrary, imatinib was able to abrogate hrSCF-mediated enrichment in spheroid-forming units (Fig. 4c). Since PDGFR-α and β are among the imatinib targets^48^, we tested their expression in a set of primary EOC samples to confirm that our results were strictly c-Kit-dependent. As shown in Fig. 4d, none of the tested samples expressed PDGFR-α and β. We next evaluated the effect of hrSCF on mRNA expression levels of stemness-associated genes such as Oct4, Sox2, and Nanog, together with c-Kit. As expected, two weeks of in vitro treatment with hrSCF induced a significant increase in the mRNA levels of all the evaluated genes (Fig. 4e). Accordingly, imatinib treatment abrogated the hrSCF-induced transcriptional regulation of these genes, bringing their mRNA levels to the baseline (Fig. 4e).

Overall, these data indicate that SCF is able to affect some of the ovarian CSC canonical features, while imatinib fully abolishes the effects operated by the cytokine.

## Discussion

The role of the microenvironment in fostering or modulating tumor growth is debated, and a wealth of data indicates that several myeloid and non-myeloid populations may positively or negatively regulate tumor development^49–51^. While the idea that interactions involving different growth factors and their cognate receptors on tumor cells could be acting in cancer has been repeatedly advanced^24,36,52–57^, as yet the evidence is faint, and mostly based on circumstantial evidence obtained in established tumor cell lines. Here, we document for the first time on ex vivo tumor cells obtained from primary EOC ascitic effusions the existence of both homotypic and heterotypic interactions between stromal, immune cells and CSC, based on a juxtacrine/paracrine circuit that involves soluble and membrane-bound SCF and its cognate receptor c-Kit, which promotes ovarian CSC survival. In particular, c-Kit expressed by ovarian CSC binds to both soluble and cell-associated SCF, expressed by tumor cells and myeloid/non-myeloid infiltrating cells, and it functionally responds to the ligand.

C-Kit is a well-known proto-oncogene as it is implicated in several human neoplasias, including small cell lung carcinoma, melanoma, testicular carcinoma, mast cell leukemia, acute myeloid leukemia, and gastrointestinal stromal tumor (GIST)^58^. Indeed, c-Kit overactivation or gain-of-function mutations in the tissues where the receptor is physiologically expressed cause their malignant transformation. The majority of the oncogenic mutations fall in the kinase domain and in the juxtamembrane domain; as a result, the receptor is constitutively activated, also in the absence of the ligand, and this phenomenon supports aberrant proliferation^16^. Moreover, Chau et al. showed that c-Kit is not just a marker of ovarian CSC, but it can also determine their stem phenotype^40^.

SCF is produced by several mesenchymal and stromal cell types^16,27^. Two isoforms of SCF exist, one secreted and one that remains membrane-anchored due to the lack of a cleaving site for metalloproteases^16,26^. We found that the soluble SCF form was only produced by TAM and TAF, whereas a minority of tumor cells only expressed the membrane-associated SCF form. In another experimental setting, Fatrai et al. reported that differentiated tumor cells from colon cancer secrete SCF, which promotes stemness features of c-Kit^+^ tumor cells in a paracrine manner^23^. We could only demonstrate the expression of membrane-anchored SCF by ovarian tumor cells; nevertheless, the findings by Fatrai were obtained in another tumor histotype. Both soluble and membrane-associated SCF are known to activate c-Kit^27,59,60^. In the present paper, it was unfeasible for obvious technical reasons to compare the strength of c-Kit and Akt activation by soluble or membrane-anchored SCF in ovarian CSC. Notwithstanding, both soluble and cell-associated SCF induced clear biochemical changes in c-Kit^+^ cells, which were affected by c-Kit inhibition. Moreover, the interaction between SCF and c-Kit entailed an increase in the canonical stemness properties of ovarian CSC, i.e., the ability to form spheroids and the expression of stemness-associated genes.

An important finding of our work is the observation that in EOC ascitic fluid SCF was expressed and produced by TAM which were M2-like. This finding is not surprising, if considering that M2 macrophages do play a fundamental regulatory role in maintaining immune system homeostasis^61^; in a tumor setting, these cells may favor the escape of tumor cells, and create an immunosuppressive *milieu*^62^. In this regard, our results are consistent with the knowledge that in the peritoneal cavity and ascites TAM are primarily M2-like and pro-tumorigenic, and associated with poor survival^63^.

To the best of our knowledge, the expression of SCF gene by functionally committed macrophages has not been investigated. In healthy donors, SCF expression was associated with monocyte activation and differentiation. However, following in vitro polarization, we obtained no clear evidence of a difference in SCF production between M1-polarized and M2-polarized macrophages.

The implications of our study are manifold. On the one hand, c-Kit expression has been repeatedly advocated as a stemness marker in several tumor histotypes, depending on the methods of detection of c-Kit^+^ cells^64–66^. Expectedly, this putative stem population could be most often below the detection limits of most histological, cytometric and molecular techniques. Without overstating our data, which focus on a single tumor where c-Kit expression is a canonical CSC marker, we wonder whether a circuit between c-Kit and SCF (whose ubiquitous production from tumor-associated stroma is reasonably expected) could represent a more widespread mechanism maintaining tumor growth. On the other hand, whether interruption of a putative SCF/c-Kit circuit could affect tumor outgrowth is a matter of debate. Imatinib is the mainstay of GIST treatment^67^, even though its efficacy is often hampered by the appearance of new mutations in c-Kit or other related tyrosine kinase receptors such as PDGFR-α^68^. More recently, imatinib administration has been extended to other tumor histotypes^69,70^. In EOC, phase II trials with imatinib as a maintenance treatment in EOC patients in complete regression after chemotherapy showed minimal activity^71–73^, a result that could be altered by the lack of EOC patient stratification according to c-Kit expression which is restricted to only 30–40% of cases^74^. Therefore, it is not far-fetched to advance that an appropriate combination of standard chemotherapy and c-Kit inhibitors in properly selected patients could be beneficial.

## Materials and methods

### Primary samples, cell lines, and in vitro culture

Ascitic effusions were obtained from 32 patients with histologically confirmed EOC. Cells were isolated by centrifugation, whereas the liquid phase was stored at −80 °C for subsequent ELISA analysis. Cells were maintained in RPMI-1640 medium (Euroclone, Milan, Italy) supplemented with 10% fetal bovine serum (FBS; GIBCO, Thermo Fisher Scientific, Waltham, MA), 100 U/mL Penicillin/Streptomycin (Lonza, Basel, Switzerland), 1 mM sodium pyruvate (Lonza), and 2 mM ultra-glutamine (Lonza). Cells were grown at 37 °C, 5% CO_2_, and harvested using trypsin-EDTA (GIBCO). Cells were maintained in adhesive conditions for about 1 week (with medium change for cell debris removal after cell attachment to the plastic). Then cells were used to prepare cell lysates or to perform sphere assay. Adherent cells were confirmed to be EOC cells by epithelial morphology and FACS analysis of CD44 marker (data not shown).

HEK293T, a human embryonic kidney cell line, and Raji, a human B-lymphoma cell line, were purchased from from American Type Culture Collection (ATCC; Manassas, VA). Kasumi-1, a human leukemic cell line, was kindly provided by Dr Francesco Piazza, (Venetian Institute of Molecular Medicine VIMM, Padova, Italy). HEK293T cells were cultured in DMEM medium (Euroclone) supplemented as described above. Raji cells were cultured in suspension in RPMI-1640 medium supplemented as described before. Kasumi-1 cells were cultured in suspension in RPMI-1640 medium, supplemented with 20% FBS, 100 U/mL Penicillin/Streptomycin, 1 mM sodium pyruvate, and 2 mM Ultraglutamine.

For spheroid-culture conditions, cells from primary samples were plated in poly-2-hydroxyethyl methacrylate (pHEMA, Sigma Aldrich, St Louis, MO)-coated non-tissue culture treated six-well plates (Corning, New York, NY) in serum-free DMEM/F12 medium (GIBCO), supplemented with 100 U/mL Penicillin/Streptomycin, 2 mM Ultraglutamine, bovine serum albumin (BSA, 4 mg/mL; Sigma Aldrich), bFGF (20 ng/mL, Peprotech, Rocky Hill, NJ), EGF (20 ng/mL, Peprotech), insulin (5 μg/mL, Sigma Aldrich), heparin (0.625 U.I/mL, PharmaTex, Milan, Italy), and B27 (GIBCO) at a density of 5 × 10^4^ cells/well. Medium was replaced every 7 days. The entire experiments lasted two weeks.

For SCF stimulation, cells were either treated with human recombinant SCF (hrSCF, 50 ng/mL, Peprotech), or cocultured with Raji-CTRL or Raji-SCF cells for 5 min (where indicated). For cocultures, a 10:1 Raji:Kasumi-1 ratio was chosen. Before stimulation, cells were pretreated or not with imatinib (30 μM, Sigma Aldrich) for 1 h 30 min at 37 °C.

### Extreme limiting dilution assay (ELDA)

To determine the frequency of spheroid-forming cells, cells were counted and plated at different concentrations in 96-well flat-bottom ultra-low attachment pHEMA-coated plates in a total volume of 0.1 ml of serum-free DMEM/F12 medium supplemented as for spheroid cultures. Fifteen replicate wells were set up for each cell concentration. After a week, the wells were scored for spheroid formation; the frequency of spheroid-forming precursors in each population was calculated by ELDA web tool (http://bioinf.wehi.edu.au/software/elda). Data are expressed as the number of spheroid-forming cells/10^3^ cells.

### Lentiviral vector production and cell transduction

For membrane-associated SCF overexpression, pLenti-C-mGFP-P2A-Puro plasmid encoding GFP-tagged human KIT ligand, transcript variant “a”, as well as the empty vector, were purchased from OriGene Technologies (Rockville, MD). One Shot™ Stbl3™ chemically competent *E. coli* (Invitrogen, Thermo Fisher Scientific) were transformed by heat shock and chloramphenicol-selected (Sigma Aldrich). Bacteria were cultured in LB broth (Sigma Aldrich), and plasmids were purified by Plasmid Maxi Kit (Qiagen, Hilden, Germany), as per manufacturer’s instructions.

Lentiviral vector stocks were generated by a transient three-plasmid vector packaging system. Briefly, HEK293T cells were co-transfected with VSV-G construct (pHCMV-G, kindly provided by Prof. Volker Erfle, Institut für Molekulare Virologie, Neuherberg, Germany), pCMVR8.74 (Addgene plasmid #22036, gift from Didier Trono, École Polytechnique Fédérale de Lausanne, Lausanne, Switzerland), and the plasmid of interest. Lentiviral particles were obtained by ultra-centrifugation of cell supernatants.

Raji cells were subjected to spinoculation: briefly, 1,000,000 cells were seeded in 24-well plates with concentrated vector-containing supernatant, centrifuged at 2400 rpm for 2 h, and incubated overnight. Then, the supernatant was replaced with complete medium. After 48 h, cells were puromycin-selected (1 μg/mL, Sigma Aldrich). Empty vector-transduced Raji cells were named Raji-CTRL; Raji cells expressing membrane SCF were named Raji-SCF.

### Flow cytometry

Cells were stained with Live/Dead fixable violet dead (1:600; Molecular Probes, Thermo Fisher Scientific) to discriminate living cells. For intracellular staining, cells were fixed with paraformaldehyde (PFA) 4%, permeabilized with Triton X-100 0.1%, and saturated with bovine serum albumin (BSA) 5% (all from Sigma-Aldrich).

The following anti-human antibodies were used: CD44 (1:1 000; Abcam, Cambridge, UK), c-Kit (1:10; Miltenyi-Biotec, Bergish Gladbach, Germany), CD45 (1:10; Miltenyi-Biotec), phospho Akt (1:100; Cell Signaling Technology, Boston, MD), SCF (1:50; Thermo Fisher Scientific), CD14 (1:20; Biolegend, San Diego, CA), CD90 (1:200; BD Bioscience, Franklin Lakes, NJ), CD3 (1:20; Miltenyi-Biotec), and CD19 (1:10; Biolegend). When needed, the secondary antibodies (Alexa Fluor, 1:500, Invitrogen, Thermo Fisher Scientific) were added.

All the cytofluorimetric analyses were performed using a FACS LSRII (BD Bioscience); data were collected from at least 1 × 10^5^ cells and elaborated with FlowJo software (TreeStar, Ashland, OR).

For FACS-sorting, antibody-labeled cells were separated with a MoFlo Astrios Cell Sorter (Beckman Coulter, Brea, CA); the purity of the sorted populations always exceeded 90%.

For the identification of the ascitic populations, the following gating strategies were used: CD45-positive cells identify cells of lympho-myeloid origin; among CD45^+^ cells, tumor-associated macrophages (TAM) were selected as CD14^+^ and tumor-infiltrating lymphocytes (TIL) as CD19^+^ (B cells) and CD3^+^ (T cells); among CD45^−^ cells, tumor-associated fibroblasts (TAF), CSC and no-CSC were selected as CD90^+^, CD44^+^c-Kit^+^, and CD44^+^c-Kit^−^, respectively.

For SCF-induced pAkt determination, after stimulation, cells were fixed in cold methanol 100%, permeabilized with Triton X-100 0.1%, blocked with FcR blocking reagent (1:5, Miltenyi Biotec), and stained with anti-phospho Akt antibody (1:33 for coculture experiment), followed by Alexa Fluor 546 goat anti-rabbit secondary antibody. P-Akt signal mean fluorescence intensity (MFI) was recorded within the GFP-negative population.

### PBMC purification, monocyte isolation, and macrophage differentiation and polarization

Peripheral blood mononuclear cells (PBMC) were isolated by density gradient centrifugation on Ficoll-Paque (GE Healthcare, Chicago, IL) from healthy donor buffy coats. Monocytes were purified from PBMC using Pan Monocyte Isolation Kit on LS Separation columns (Miltenyi-Biotec). Monocytes were cultured at a density of 1 × 10^6^ cells/mL for 7 days in FBS-coated dishes in RPMI-1640 medium supplemented with 20% FBS, in the presence of granulocyte-macrophage colony-stimulating factor (GM-CSF, 100 ng/mL, Peprotech) for differentiation into M0 macrophages. Subsequently, M0 macrophages were stimulated with LPS (100 ng/mL; Sigma Aldrich) and IFN-γ (20 ng/mL; Peprotech) for M1 polarization, and with IL-4 (20 ng/mL; Peprotech) and IL-13 (20 ng/mL; Peprotech) for M2 polarization, in RPMI-1640 medium supplemented with 5% FBS, as reported elsewhere^75^. After 24 h and 48 h, the conditioned medium was collected, 50-fold concentrated with Amicon^®^ Ultra-15 centrifugal filter units (Merck Millipore, Sigma Aldrich) and stored at −80 °C for subsequent ELISA analysis, while cells were harvested for flow cytometry, Western blot and RNA extraction. M1 and M2 polarization was confirmed by qRT-PCR analysis of M1 (IL-1β and TNF-α)^76^ and M2 (CCL22 and IL-10) marker^77^ expression.

### Immunofluorescence (IF)

Monocytes were either fixed in tubes as suspension cells or seeded on coverslips and then differentiated into M0 macrophages. Cells were fixed in PBS 3.7% formaldehyde (Sigma Aldrich), permeabilized with PBS 0.1% Triton X-100, and blocked with PBS 10% FBS. The anti-human SCF primary antibody (1:50, Thermo Fisher Scientific) was incubated overnight at 4 °C in PBS 10% FBS; subsequently, the Alexa Fluor 488-conjugated secondary antibody (1:500) and the F-actin-binding Alexa Fluor 594-conjugated Phalloidin (1:150, Molecular Probes, Thermo Fisher Scientific) were added for 2 h at room temperature. Nuclei were stained with DAPI (1:500, Invitrogen) and coverslips were mounted by using Fluorescent Mounting Medium (DAKO, Jena, Germany). Samples were analyzed with a Leica TCS SP5 inverted laser scanning confocal microscope (Leica, Wetzlar, Germany) equipped with a Leica HCX PL APO lambda blue ×63/1.40 oil UV objective and standard PMT detectors. Z-sectioning was performed with the Leica LAS AF software in order to produce an image with an original resolution of 1024 × 1024 pixels every micron of the whole sample thickness. Finally, maximum projections were generated from z-stacks. In the shown images the display lookup table (LUT) was linear and covered the full range of the data.

### Enzyme-linked immunosorbent assay (ELISA)

Ex vivo ascitic liquid phases, concentrated conditioned media from M0, M1, and M2 polarized macrophages, and FACS-separated CSC, noCSC, TAF, TAM, and TIL ascitic cell population supernatants were tested for soluble SCF production with a Human SCF Quantikine^®^ ELISA Kit (R&D, Minneapolis, MN), according to manufacturer’s instructions. For all the different cell subtypes, cells were plated at a concentration of 1 × 10^6^ cells/mL and medium was collected after 24 h of culture.

### Western blotting (WB)

Cells were lysed with RIPA buffer supplemented with protease and phosphatase inhibitors. Protein concentration was determined by using the bicinchoninic acid (BCA) assay (Quantum Micro Protein, Euroclone). Equal protein amounts were loaded on NuPAGE™ 4–12% Bis-Tris protein precast polyacrylamide gels (Invitrogen, Thermo Fisher Scientific) in denaturing and reducing conditions. Proteins were then transferred onto nitrocellulose membranes (Perkin Elmer, Waltham, MA). Membranes were saturated with 5% non-fat milk in TBS-Tween 20 buffer, and hybridized overnight at 4 °C with the following primary anti-human antibodies: β-actin (1:1000; Abcam), Akt (1:1000; Cell Signaling Technology), phospho Akt (1:2000; Cell Signaling Technology), c-Kit (1:1000; Cell Signaling Technology), and phospho c-Kit (1:1000; Cell Signaling Technology). Primary antibodies were diluted either in 5% milk or in 5% BSA in TBS-Tween 20 buffer, depending on manufacturer’s instructions. Secondary horseradish peroxidase (HRP)-conjugated anti-rabbit or anti-mouse antibodies (Perkin Elmer), diluted 1:5000 in 1% milk in TBS-Tween 20 buffer, were added for 1 h at room temperature. Finally, the chemiluminescence signal was detected with Western Lightning^®^ Plus-ECL (Perkin Elmer) on a ChemiDoc™ XRS Imaging System (Bio-Rad, Hercules, CA), and band densitometry was analyzed by Quantity One^®^ software (Bio-Rad). Signal intensity was normalized to β-actin.

### RNA extraction, reverse transcription, and quantitative PCR

Total RNA was extracted by the TRIzol method according to manufacturer’s instructions (Ambion, Thermo Fisher Scientific). cDNA was synthesized from 1 μg of total RNA using the High capacity RNA-to-cDNA kit (Applied Biosystems, Thermo Fisher Scientific), then it was mixed with the gene-specific primers and Platinum™ SYBR™ Green qPCR SuperMix-UDG (Invitrogen, Thermo Fisher Scientific); each sample was run in duplicate. The PCR step was performed using an ABI PRISM® 7900HT Sequence Detection System (Applied Biosystems, Thermo Fisher Scientific). Results were analyzed using the comparative ΔΔCt method; ΔΔCt values were utilized to calculate the RQ = 2^−ΔΔCt^. Data were expressed as the fold difference in gene expression (normalized to the housekeeping gene β_2_-microglobulin) relative to a reference sample, as indicated in the individual figure legends.

Primers were purchased from Sigma Aldrich. Primer sequences are reported in Supplementary Table 2.

### SCF coating experiment

Twenty-four-well plates were coated with hrSCF (5 μg/mL) or BSA (5 μg/mL, negative control) dissolved in carbonate-bicarbonate buffer pH 9.6, overnight at 4 °C. The day after, wells were washed with PBS and cells were added on top. Plates were then spun for 2 min at 1300 rpm at RT to allow contact with plate bottom and incubated for 5 min at 37 °C. Cells were then collected and lysed for protein extraction. Where indicated, cells were pretreated with 30 μM imatinib for 1 h 30 min at 37 °C.

### Statistical analysis

Data from replicate experiments were shown as mean values ± SD. Comparisons between groups were done by the two-tailed Student’s *t*-test and Mann–Whitney test, as appropriate. A *P* value < 0.05 was considered as significant.

### Study approval

This study was approved by the local Institutional Review Board and Ethics Committee, and conforms to the rules of the Declaration of Helsinki. Written informed consent was obtained from all participants entering this study.

## Supplementary information

Supplementary information.

Supplementary Table 1.

Supplementary Table 2.

Supplementary Figure 1.

Supplementary Figure 2.

Supplementary Figure 3.
