# Supplementary material for: A juxtacrine/paracrine loop between C-Kit and stem cell factor promotes cancer stem cell survival in epithelial ovarian cancer
Source: Cell Death Dis. 2019 May 28;10(6):412. doi: 10.1038/s41419-019-1656-4 (PMC6538673; doi:10.1038/s41419-019-1656-4)
Supplement: Supplementary file 1 — Supplementary information. [file 41419_2019_1656_MOESM1_ESM.docx]

**Supplementary information**

A JUXTACRINE/PARACRINE LOOP BETWEEN C-KIT AND STEM CELL FACTOR PROMOTES CANCER STEM CELL SURVIVAL IN EPITHELIAL OVARIAN CANCER

Mazzoldi Elena Laura, Pavan Simona, Pilotto Giorgia, Leone Kevin, Pagotto Anna, Frezzini Simona, Nicoletto Maria Ornella, Amadori Alberto, and Pastò Anna

**Supplementary figure legends**

**Supplementary Figure S1.** M1 and M2 marker analysis in *in vitro* polarized macrophages. M1 (IL-1β and TNF-α) and M2 (CCL22 and IL-10) markers were analyzed by qRT-PCR in M0, M1, and M2 macrophages after 24h **(A)** and 48h **(B)** from polarization. The bars represent the mean ± S.D. (N=3). Data were normalized to M0.

**Supplementary Figure S2.** SCF isoform expression in M0, M1, and M2 macrophages. SCF 220 and SCF 248 were analyzed by qRT-PCR after 6h, 9h, 16h, and 24h after M0 polarization with LPS/IFNγ (M1) and IL-4/IL-13 (M2). Data were normalized to M0.

**Supplementary Figure S3.** SCF 220 (GFP-tagged) expression in Raji cells. SCF 220 (GFP-tagged) expression was checked by Western blot **(A)** and qRT-PCR **(B)** analyses in Raji cells after transfection and selection. Raji-CTRL expressing only GFP were used as a control. N.D. = not detectable.
