# Supplementary material for: A juxtacrine/paracrine loop between C-Kit and stem cell factor promotes cancer stem cell survival in epithelial ovarian cancer
Source: Cell Death Dis. 2019 May 28;10(6):412. doi: 10.1038/s41419-019-1656-4 (PMC6538673; doi:10.1038/s41419-019-1656-4)
Supplement: Supplementary file 2 — Supplementary Table 1. [file 41419_2019_1656_MOESM2_ESM.docx]

**Supplementary Table 1.** SCF concentration in EOC patient ascites does not correlate with any ovarian tumor histological subtypes, stage and grade.

|  | **N° samples** | **SCF pg/ml (median ± SD)** | **P-value** |
| --- | --- | --- | --- |
| **HISTOLOGY** |  |  |  |
| Endometrioid^1^ | 1 | 1 953.5 |  |
| serous tubal^1^ | 2 | 1 153.1 ± 499.1 |  |
| undifferentiated | 6 | 1 105.1 ± 249.4 | 0.083^2^ |
| serous papillary | 13 | 1 599.2 ± 442.2 |  |
| serous | 6 | 1 102.7 ± 353.6 |  |
| **STAGING** |  |  |  |
| stage 3 | 17 | 1 385.7 ± 441.3 | 0.161^3^ |
| stage 4 | 9 | 1 107.7 ± 354.8 |  |
| **GRADING** |  |  |  |
| G1^1^ | 1 | 1 338.4 |  |
| G3 | 19 | 1 371 ± 455.2 | 0.192^3^ |
| G4 | 6 | 1 105 ± 249.4 |  |

^1^ Not used for statistical analysis

^2^ Kruskal-Wallis test was used

^3^ Mann-Whitney test was used
