# Supplementary material for: A juxtacrine/paracrine loop between C-Kit and stem cell factor promotes cancer stem cell survival in epithelial ovarian cancer
Source: Cell Death Dis. 2019 May 28;10(6):412. doi: 10.1038/s41419-019-1656-4 (PMC6538673; doi:10.1038/s41419-019-1656-4)
Supplement: Supplementary file 3 — Supplementary Table 2. [file 41419_2019_1656_MOESM3_ESM.docx]

**Supplementary Table 2.** Primers used for qRT-PCR.

| Gene Name | Gene Symbol | GenBank | Forward | Reverse |
| --- | --- | --- | --- | --- |
| β2micro | B2M | NM_004048 | 5’-TCTCTCTTTCTGGCCTGGAG-3’ | 5’-TCTCTGCTGGATGACGTGAG-3’ |
| SCF 248 | KITLG | NM_000899 | 5’-CCATTGATGCCTTCAAGGAC-3’ | 5’-TGGCCTTCCTATTACTGCTACT-3’ |
| SCF 220 | KITLG | NM_003994 | 5’-CTGAGAAAGGGAAGGCCAA-3’ | 5’-GGCTCCAAAAGCAAAGCCAA-3’ |
| IL-1β | IL1B | NM_000576 | 5’-TGAAAGCTCTCCACCTCCAG-3’ | 5’-GCCCAAGGCCACAGGTATTT-3’ |
| iNOS | NOS2 | NM_000625 | 5’-CCACCAGTATGCAATGAATGGG-3’ | 5’-TGCTGCTTGCTGAGGTTGTGAT-3’ |
| TNF-α | TNF | NM_000594 | 5’-GGACCTCTCTCTAATCAGCC-3’ | 5’-GGGTTTGCTACAACATGGGC-3’ |
| ARG2 | ARG2 | NM_001172 | 5’-TGACATCAACACACCCCTTACC-3’ | 5’-GTCCACGTCTCTCAGACCAAT-3’ |
| IL-10 | IL10 | NM_000572 | 5’-TGCTGGAGGACTTTAAGGGT-3’ | 5’-CGCCTTGATGTCTGGGTCTT-3’ |
| CCL22 | CCL22 | NM_002990 | 5’-GAAACACTTCTACTGGACCTCA-3’ | 5’-AATCATCTTCACCCAGGGCA-3’ |
| OCT4 | POU5F1 | NM_002701 | 5’-GAAGGATGTGGTCCGAGTGT-3’ | 5’-CCTCTCGTTGTGCATAGTCG-3’ |
| SOX2 | SOX2 | NM_003106 | 5'-CAAGATGCACAACTCGGAGA-3' | 5'-GCTTAGCCTCGTCGATGAAC-3’ |
| NANOG | NANOG | NM_024865 | 5’-AGATGCCTCACACGGAGACT-3’ | 5’-AGGGCTGTCCTGAATAAGCA-3’ |
| c-KIT | KIT | NM_000222 | 5’-GGATTCCCAGAGCCCACAAT-3’ | 5’-GGCAGTACAGAAGCAGAGCA-3’ |
