# Supplementary figures and images for: A juxtacrine/paracrine loop between C-Kit and stem cell factor promotes cancer stem cell survival in epithelial ovarian cancer
Source: Cell Death Dis. 2019 May 28;10(6):412. doi: 10.1038/s41419-019-1656-4 (PMC6538673; doi:10.1038/s41419-019-1656-4)

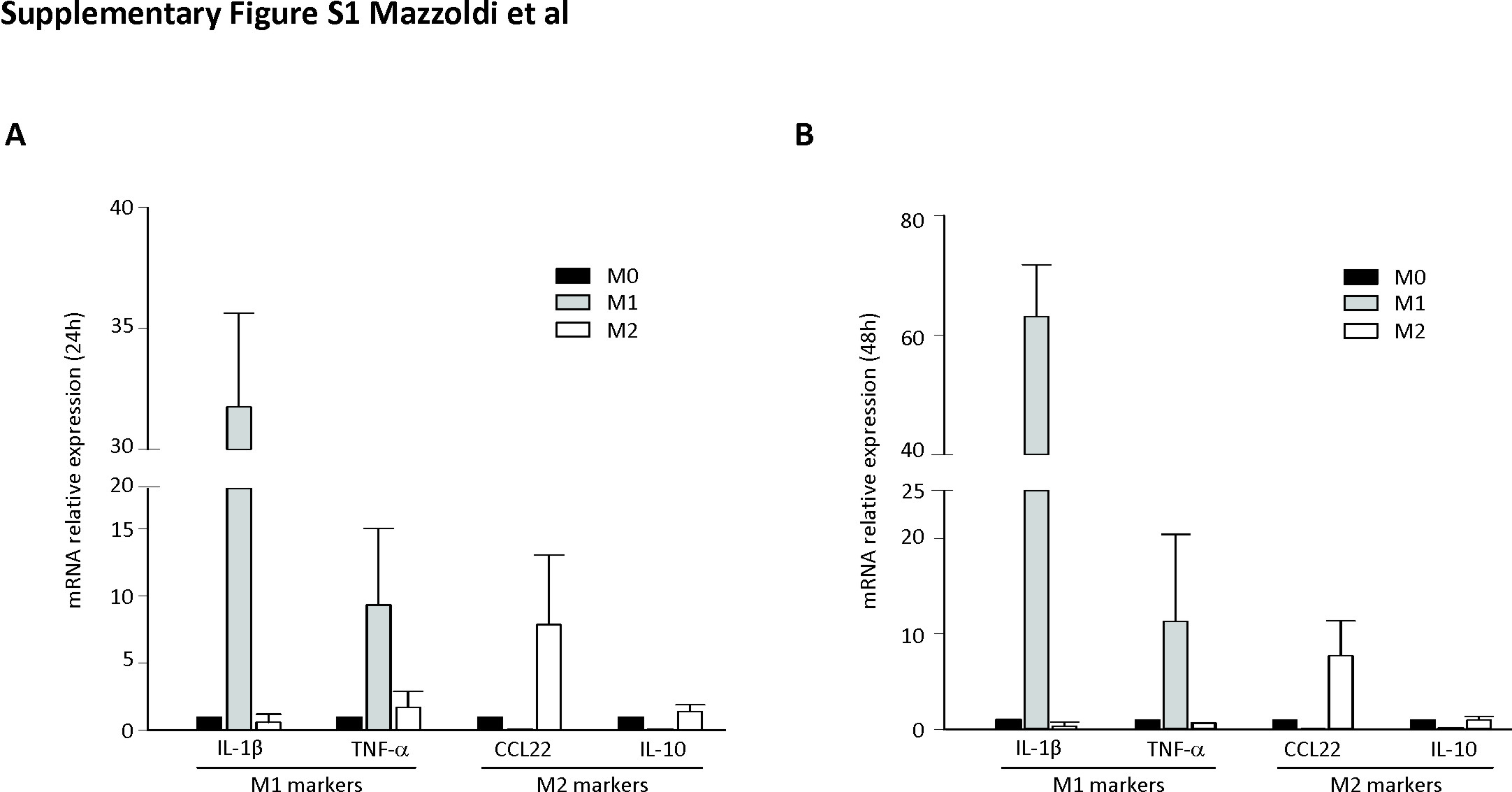

Supplement: Supplementary file 4 — Supplementary Figure 1. [file 41419_2019_1656_MOESM4_ESM.jpg]

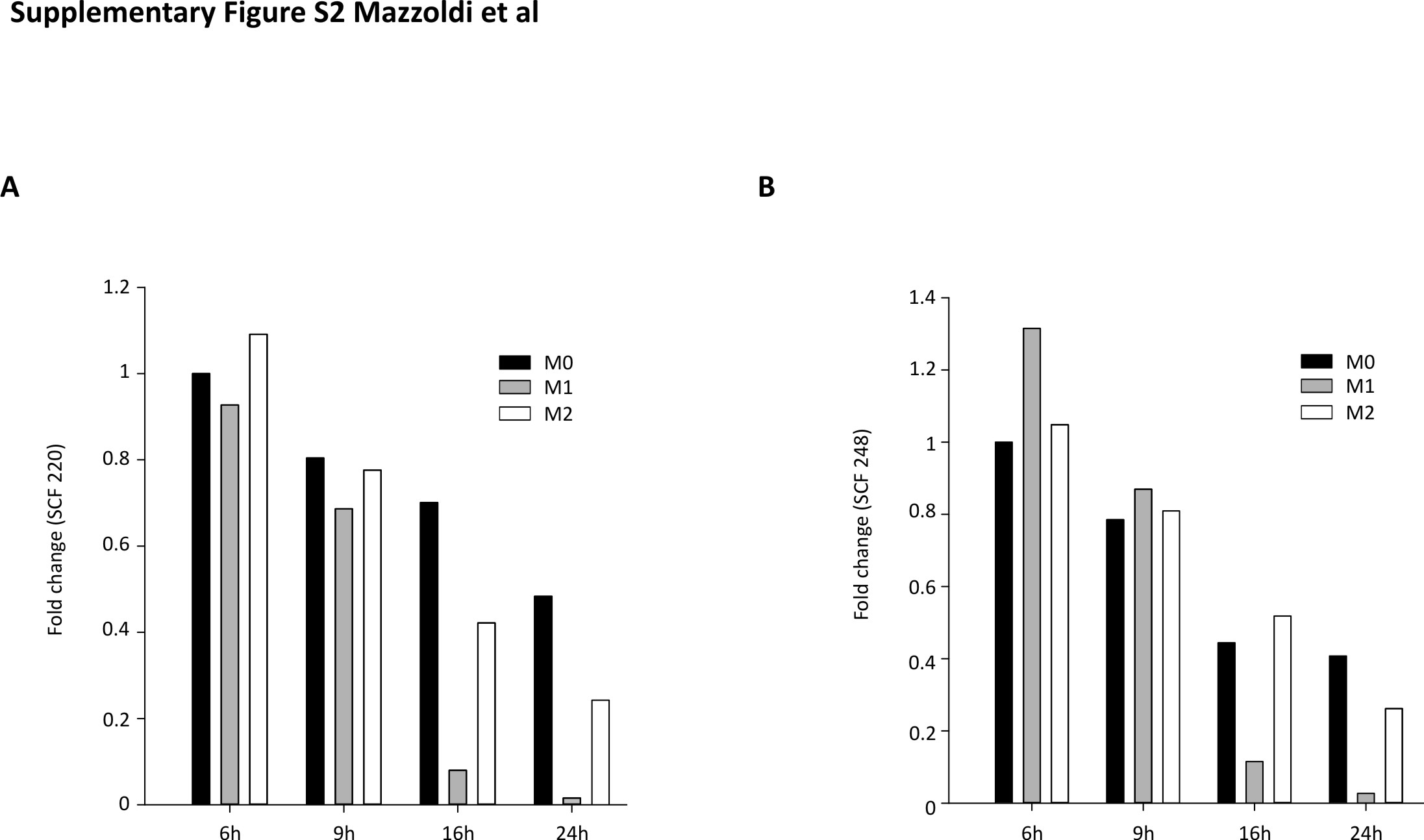

Supplement: Supplementary file 5 — Supplementary Figure 2. [file 41419_2019_1656_MOESM5_ESM.jpg]

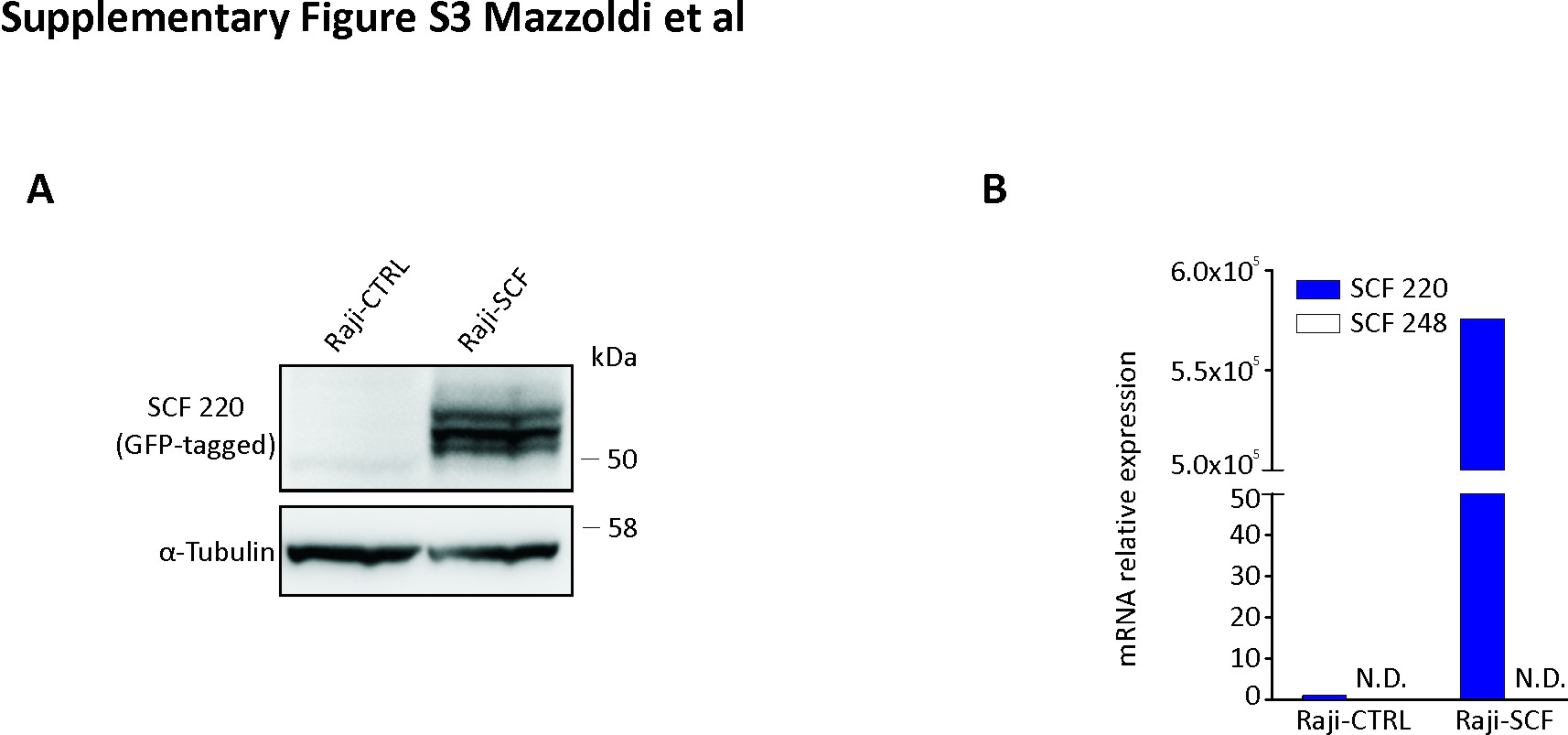

Supplement: Supplementary file 6 — Supplementary Figure 3. [file 41419_2019_1656_MOESM6_ESM.jpg]
